# Copper metabolism-related biomarkers and therapeutic targets for diabetic nephropathy

**DOI:** 10.7717/peerj.20468

**Published:** 2025-12-19

**Authors:** Qiaofang Yan, Yuanyuan Du, Fei Huang, Min Zhan, Qifan Zheng, Qiaoxuan Zhang, Pengwei Zhang, Jun Yan, Xiaobin Wu, Haibiao Lin, Xianzhang Huang, Liqiao Han

**Affiliations:** 1 The Second Affiliated Hospital of Guangzhou University of Chinese Medicine, Guangzhou, China; 2 Second Clinical Medical College, Guangzhou University of Chinese Medicine, Guangzhou, China; 3 Shenzhen Traditional Chinese Medicine Hospital, Shenzhen, Guangdong, China; 4 Beibei District Traditional Chinese Medicine Hospital, Chongqing, China

**Keywords:** Diabetic nephropathy, Copper metabolism, Biomarker, Immune infiltration

## Abstract

**Background:**

Diabetic nephropathy (DN) is the most intractable complication of diabetes. Despite decades of research, accurate diagnostic markers and effective therapeutic drugs are still elusive. Abnormal copper metabolism is also implicated in diabetes and its complications. This study aims to identify copper metabolism-related biomarkers and potential drugs for DN.

**Methods:**

DN datasets and copper metabolism-related genes (CMGs) were obtained from Gene Expression Omnibus (GEO) and GeneCards. Differentially expressed CMGs (DE-CMGs) were identified using the limma package and the Venn algorithm. Functional enrichment analysis and protein-protein interaction (PPI) network were performed to identify candidate hub genes. The single gene with an area under the receiver operating characteristic (ROC) curve > 0.7 was identified as a potential diagnostic biomarker of DN. Finally, these biomarkers were validated by quantitative real-time polymerase chain reaction (qRT-PCR) in high-glucose-treated human proximal tubular (HK-2) cells. These validated hub genes were used to construct a combined prediction model, confirmed by additional GSE30528 and GSE30529 datasets. The correlation analysis between the expression level of the hub genes and the estimated glomerular filtration rate (eGFR) was carried out. Additionally, immune cell infiltration and potential target drugs were investigated for these biomarkers.

**Results:**

Five hub genes associated with copper metabolism, namely CD36, CCL2, CASP3, LPL, and APOC3, were identified as biomarkers for the early diagnosis of DN. Utilizing multiple biomarkers enhanced diagnostic accuracy and specificity. CD36, CCL2, and CASP3 correlated negatively with eGFR levels, while LPL and APOC3 correlated positively. Additionally, these hub genes were significantly linked to various immune cell types, including macrophages M1 and M2, T cells, gamma delta resting dendritic cells, neutrophils, and NK cells. Furthermore, 15 agents targeting these biomarkers were retrieved from the DrugBank database.

**Conclusion:**

Our study identified key genes possibly related to copper metabolism in the pathological mechanism of DN that could serve as novel targets for the diagnosis and therapy of DN.

## Introduction

Diabetic nephropathy (DN), the most common complication of diabetes, represents the primary cause of end-stage renal disease (ESRD) (Schmidt, 2023). It affects nearly one-third of all diabetic patients and is associated with a ten-year cumulative mortality rate as high as 31.1% (Yao et al., 2022). Early detection of DN is imperative to prevent its progression into renal failure. Generally, the early clinical manifestations of DN are present through glomerular hyperfiltration rate and microalbuminuria (Giralt-López et al., 2020), yet a subset of diabetic patients progresses to ESRD without notable albuminuria (Ali et al., 2022). Treatment strategies targeting blood pressure, glucose levels, and the renin-angiotensin system (RAS) are employed to slow disease progression. However, their effectiveness varies due to the heterogeneous nature of DN, underscoring the urgent need to further elucidate its underlying pathogenesis.

The pathogenesis of DN is complex and involves various factors such as glucose and lipid metabolism disorders, changes in hemodynamics, oxidative stress, inflammation, cytokine activity, and their interactions (Li et al., 2022). Moreover, oxidative stress plays a significant role in the development of DN by facilitating glomerular endothelial cell damage, and renal fibrosis (Xie et al., 2024). Multiple studies have indicated that anomalies in copper metabolism can lead to oxidative stress by Fenton-like reactions in DN, producing reactive hydroxyl radicals (Rivera-Millot, Harrison & Veyrier, 2023; Rivera et al., 2021). Meanwhile, genetic models of copper homeostasis dysregulation share cuproptosis, a newly identified cell death regulatory mechanism (Tsvetkov et al., 2022). Cuproptosis, a copper-dependent cell death mechanism involving ferredoxin 1 (FDX1) and lipoylated proteins, may exacerbate renal injury in DN by disrupting mitochondrial function. Disruptions in copper metabolism impact lipid metabolism and trigger an inflammatory response (Zhang et al., 2024). Consequently, understanding copper metabolism can enhance our comprehension of the disease and contribute to the development of treatments.

The complex relationship between abnormal copper metabolism and gene expression regulation has been studied in various diseases, including Wilson’s disease, Alzheimer’s disease, obesity, hypertension, and cancer (Du et al., 2023; Gu et al., 2020; Ge et al., 2022). Abnormal copper metabolism has been implicated in the pathogenesis of DN (Zou et al., 2024). Copper dysregulation has been identified in DN animal models, but human studies linking specific copper metabolism-related genes (CMGs) to DN progression are limited. These findings underscore the significance of exploring the potential consequences of genes related to copper metabolism in the onset and progression of DN. Traditional biomarkers, such as microalbuminuria and serum creatinine, have been shown to lack adequate sensitivity and specificity in detecting early kidney injury (Rivera-Millot, Harrison & Veyrier, 2023; Radcliffe et al., 2017), highlighting the need for novel biomarkers with higher diagnostic accuracy for DN. Copper metabolism-related genes could provide valuable insights into the diagnosis and preventive strategies for DN. Targeting copper metabolism could modulate oxidative stress and inflammation, offering a novel therapeutic avenue for DN management. Therefore, our study aims to identify pivotal copper metabolism-related genes of DN and then to predict potential treatment targets.

In this study, we employed an integrated bioinformatics approach to screen for differentially expressed CMGs and identify hub genes, followed by experimental validation in vitro. The diagnostic potential of the identified biomarkers was further assessed using independent datasets. Furthermore, the immune infiltration landscape and potential therapeutic agents associated with these key genes were explored. These findings offer novel insights into the molecular mechanisms underlying the pathogenesis of DN and may facilitate the discovery of potential biomarkers and therapeutic targets.

## Methods

This study employed a multi-step bioinformatics and experimental approach to identify copper metabolism-related biomarkers for DN, integrating transcriptomic analysis, functional enrichment, protein interactions, immune infiltration, and *in vitro* validation.

### Obtainment of DN-associated microarray datasets

Gene Expression Omnibus (GEO) is a public genomics data repository created and maintained by the National Center for Biotechnology Information (NCBI) (https://www.ncbi.nlm.nih.gov/geo/), which contains high-throughput gene expression data, chips and microarrays. Gene expression data of DN patients were selected using the search keyword “diabetic nephropathy” based on the following inclusion criteria: (1) organism: Homo sapiens; (2) study type: expression profiling by array; and (3) samples: each dataset included tubules and glomeruli tissue samples, with the largest possible sample size. Ultimately, three microarray datasets meeting the inclusion criteria were obtained from the GEO database for analysis of differential mRNA expressions. The GSE30122 dataset, used to identify differentially expressed genes (DEGs), comprises 19 DN patients and 50 age-matched normal controls. The GSE30528 and GSE30529 datasets were utilized for model validation in subsequent analyses. The batch effects were eliminated by employing the surrogate variable analysis (SVA) algorithm in R software (Fig. S1). Details of the data sample collection are outlined in Table 1.

**Table 1:** Detailed datasets information.

Dataset	Samples	Source	Platform/Technology
GSE30122	69 (19DN, 50 NC)	Glomeruli, Tubuli	GPL571 [HG-U133A_2] Affymetrix Human Genome U133A 2.0 Array
GSE30528	22 (9DN, 13 NC)	Glomeruli	GPL571 [HG-U133A_2] Affymetrix Human Genome U133A 2.0 Array
GSE30529	22 (10DN, 12 NC)	Tubuli	GPL571 [HG-U133A_2] Affymetrix Human Genome U133A 2.0 Array

**Notes.**

DN diabetic nephropathy NC normal control

### Acquisition of copper metabolism-related genes

In this study, relevant genes were identified by querying “copper metabolism” as a keyword in GeneCards (https://www.genecards.org/), a comprehensive human gene database. The degree of correlation between these genes and copper metabolism was assessed using correlation coefficients, which range from 0 to 100. Genes with a score exceeding 10 were classified as copper metabolism-related genes, this threshold excludes housekeeping genes and low-confidence entries, ensuring the analyzed genes are directly relevant to copper metabolism, thereby enabling further analysis (Du et al., 2023; Yuan et al., 2023; Yang, Wang & Zhang, 2025).

### Identification of DE-CMGs in DN

Following the predetermined statistical threshold of —log2 Fold Change —>0.5 and *P*-values < 0.05, DEGs were identified utilizing the limma package in R software (version 4.3.1). To visually depict the expression patterns of these DEGs, volcano plots and heatmaps were generated using R. Moreover, to pinpoint the genes that are both differentially expressed and correlated with copper metabolism, a Venn diagram was constructed to visualize the overlap of results.

### Enrichment analysis on DE-CMGs

The integrative approach allowed for a comprehensive exploration of the functional roles and pathways associated with DE-CMGs. Two distinct enrichment analysis tools were employed, each utilizing different algorithms to ensure robustness. Gene Ontology (GO) analysis, a widely-used bioinformatics approach, was utilized to investigate the functional roles of genes in terms of biological processes (BP), molecular functions (MF), and cellular components (CC) (Ashburner et al., 2000). Additionally, Kyoto Encyclopedia of Genes and Genomes (KEGG) analysis was employed to gain insights into advanced biological functions through genome sequencing and other high-throughput methods (Kanehisa & Goto, 2000).

### Protein-protein interaction networks and identification of hub genes

To identify interacting DE-CMGs, the PPI network was constructed based on the STRING database (https://cn.string-db.org/). PPI pairs with a combined confidence score (>0.4) were extracted and visualized within the network. Subsequently, hub genes were identified using the cytoHubba plugin in conjunction with the MCC (Maximal Clique Centrality) algorithm implemented in Cytoscape software, leveraging the established PPI network.

### Immune landscape

Immune cell infiltration was assessed using CIBERSORTx, a computational tool equipped with 547 biomarkers and capable of characterizing 22 human immune cell types, spanning myeloid subpopulations, natural killer (NK) cells, plasma cells, as well as naive and memory B and T cell subsets. Employing a linear support vector regression principle, CIBERSORTx facilitated the deconvolution analysis of expression matrices of immune cells. In this study, expression data from GSE30122 was utilized to quantify the relative proportions of the 22 immune cell types in each sample. The resulting findings were visually represented through heatmaps and boxplots.

### Cell culture

HK-2 cells, a renowned renal tubular epithelial cell line, were obtained from the National Collection of Authenticated Cell Cultures (catalog number: SCSP-511, identifier: CSTR:19375.09.3101HUMCSP511) and used between passages seven and 12 to ensure consistent cell phenotype and experimental stability. They were cultured in keratinocyte-SFM medium including epidermal growth factor, bovine pituitary extracts, and 1% serum (Invitrogen, CA) at 37 °C with 5% CO2. In the experiment, HK-2 cells were exposed to two distinct glucose conditions: high glucose (HG; 30 mM glucose) or normal glucose (NG; 5.5 mM glucose + 24.5 mM mannitol) for 48 h (Xue et al., 2021). Next, cells from both the high glucose and normal glucose groups were submitted for quantitative real-time PCR (qRT-PCR).

### Total RNA extraction and qRT-PCR

Following cell treatment, total RNA was isolated from two groups of HK-2 cells using TRIzol^®^ reagent (Invitrogen; Thermo Fisher Scientific, Inc., Waltham, MA, USA) according to the manufacturer’s protocols. The concentration and purity of the extracted total RNA were assessed using an ultraviolet spectrophotometer (NanoDrop2000, Thermo Fisher Scientific, Waltham, MA, USA), and one µg was subsequently reverse-transcribed to cDNA. The reverse-transcribed reaction conditions included 37 °C for 15 min, 85 °C for 5 s and stored at 4 °C. cDNA was stored at −20 °C and used for further experiments in a week. qRT-PCR was conducted on a Roche Z480 Real-Time PCR system (Roche Molecular Diagnostics, Indianapolis, IN, USA) employing the SYBR-Green PCR kit (Takara Bio, Inc., Shiga, Japan). The PCR conditions included a denaturing step at 95 °C for 30 s and 40 cycles of 95 °C for 5 s and 60 °C for 30 s for real time plate read. The melt curve stage included 95 °C for 5 s, 60 °C for 1 min and 95 °C for 1 s. mRNA expression levels were normalized using glyceraldehyde 3-phosphate dehydrogenase (GAPDH) as an internal control. Comparative quantification was determined utilizing the 2^−ΔΔCq^ method (Livak & Schmittgen, 2001). Samples were prepared and analyzed in triplicate, and the results are presented as the mean ± standard deviation (SD) for comparative analysis. Primer sequences used in the study are listed in Table 2.

### Construction and validation of the diagnostic model

To develop a diagnostic model for DN classification, a logistic regression algorithm was employed based on the combination of validated key genes. The area under the receiver operating characteristic curve (AUC) was calculated to assess the discriminative performance of the model, utilizing the “ROCR” package for analysis. Subsequently, the performance of the diagnostic model was further validated using the combined GSE30528 and GSE30529 dataset.

### Prediction of potential therapeutic agents

The DrugBank database (https://go.drugbank.com/) serves as a vast repository of bioinformatics and cheminformatics, containing comprehensive drug data alongside extensive drug target information. With a repertoire exceeding 7,800 drugs, it encompasses FDA-approved small-molecule drugs, biotech drugs, nutraceuticals, and experimental drugs. Leveraging the resources of DrugBank, this research aimed to predict potential therapeutic agents for DN.

### Clinical analysis

The Nephroseq v5 database (https://nephroseq.org/), a comprehensive information platform for gene expression datasets of kidney diseases, was utilized to validate the correlation between the hub genes and clinical manifestations of DN by Pearson’s correlation analysis.

**Table 2:** Primer sequences used for gene expression studies.

Gene name	Forward (5′–3′)	Reverse (5′–3′)
GAPDH	GCACCGTCAAGGCTGAGAAC	TGGTGAAGACGCCAGTGGA
CD36	GGCTGTGACCGGAACTGTG	AGGTCTCCAACTGGCATTAGAA
CCL2	GAATCACCAGCAGCAAGTGTC	CGGAGTTTGGGTTTGCTTGTC
CASP3	TGCTATTGTGAGGCGGTTGT	TCTGTTGCCACCTTTCGGTT
LPL	GGGAGTTTGGCTCCAGAGTTT	TGTGTCTTCAGGGGTCCTTAG
APOC3	GTTACATGAAGCACGCCACC	CACGGCTGAAGTTGGTCTGA
CETP	GCCAAGGTAGCTTTCCAGGAT	TGATGTCGAAGAGGCTCACG

### Statistical analysis

Statistical analysis was carried out using R software (version 4.1.0) or GraphPad Prism 8.0 software (GraphPad Software, Inc., La Jolla, CA, USA). Unpaired, two-tailed t-tests were employed to compare two experimental groups, with probability (*P*) values less than 0.05 considered statistically significant.

## Results

This study identified copper metabolism-related biomarkers for DN through integrated bioinformatics and experimental analyses, as detailed below.

### Screening for DE-CMGs

**Figure 1:**
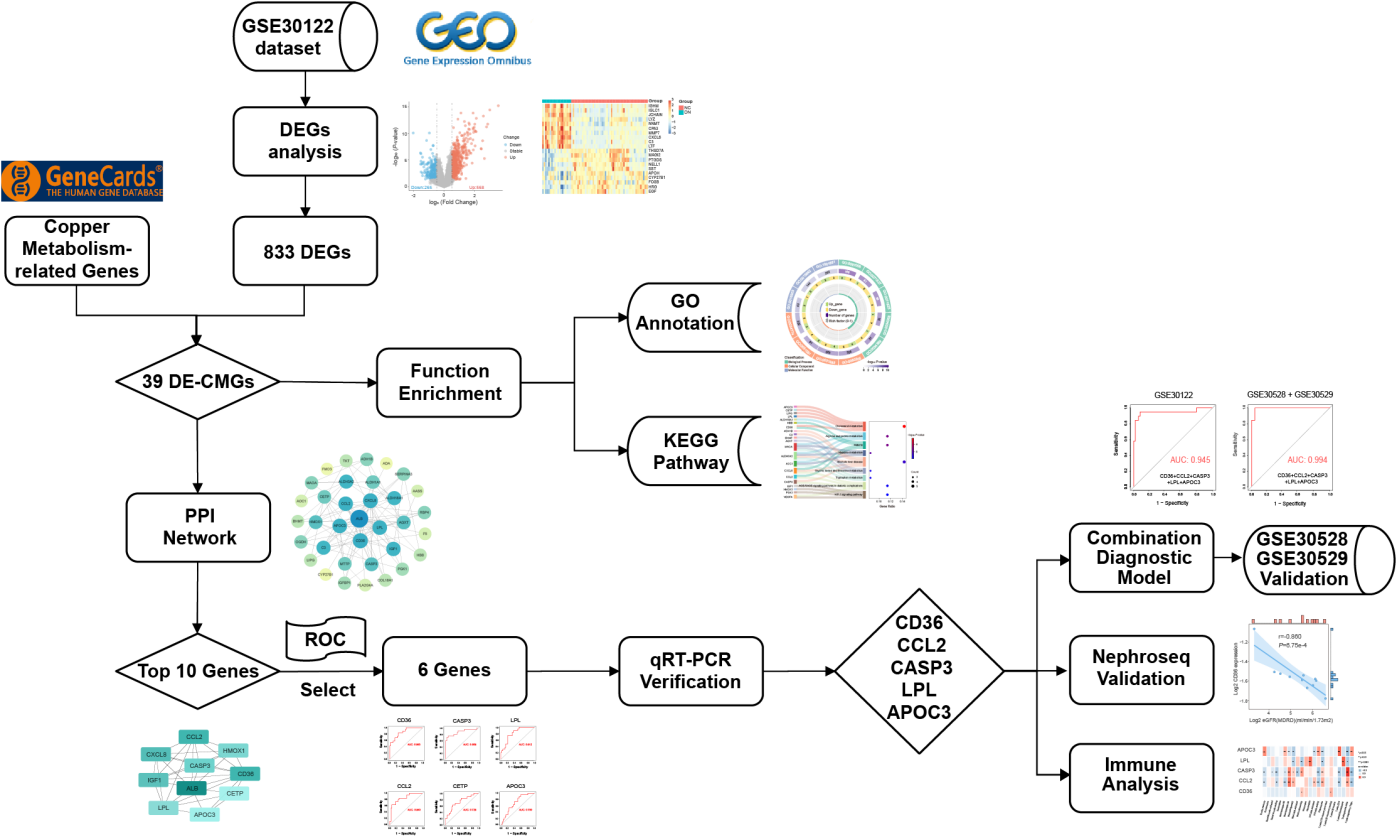
Flowchart for this study.

**Figure 2:**
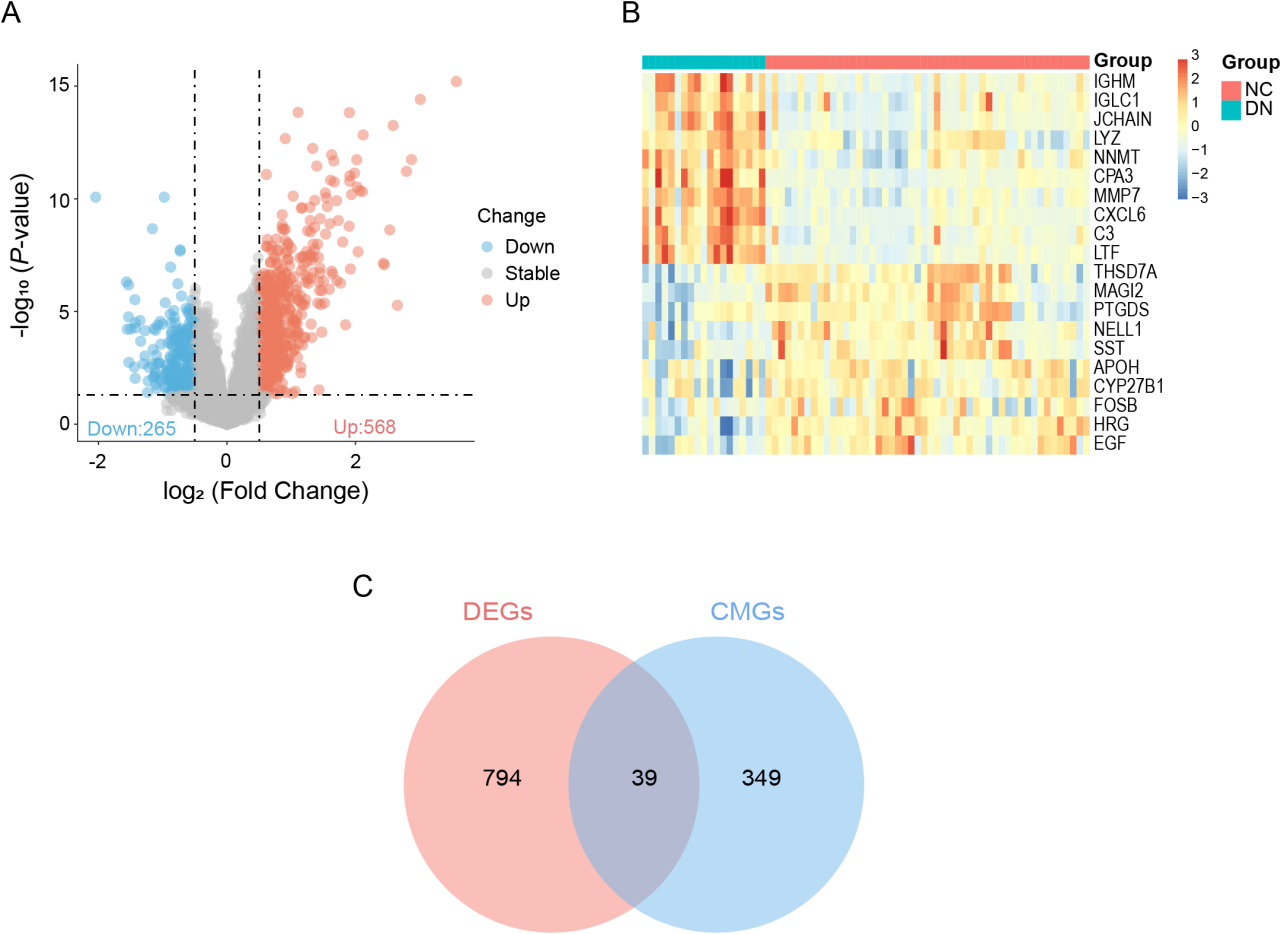
Identification of 39 DE-CMGs. (A) Volcano plot illustrating significant genes from limma analysis (blue: downregulated; red: upregulated; gray: no significant alteration). (B) Top 20 DEGs expression heatmap for DN and control groups. (C) 39 DE-CMGs were obtained by taking the intersections of the DEGs and CMGs. DE-CMGs, differentially expressed copper metabolism-related genes; DEGs, differentially expressed genes; CMGs, copper metabolism-related genes; DN, diabetic nephropathy; NC, normal control.

The study flowchart is presented in Fig. 1. A total of 833 differentially expressed genes (DEGs) were identified between DN and normal controls in the GSE30122 dataset, as visualized in a volcano plot (Fig. 2A). The top 20 DEGs are shown by heatmaps (Fig. 2B). Moreover, when comparing the DEGs with the 388 CMGs obtained from GeneCards, 39 DE-CMGs were found to overlap (Fig. 2C).

### Enrichment analysis of DE-CMGs

**Figure 3:**
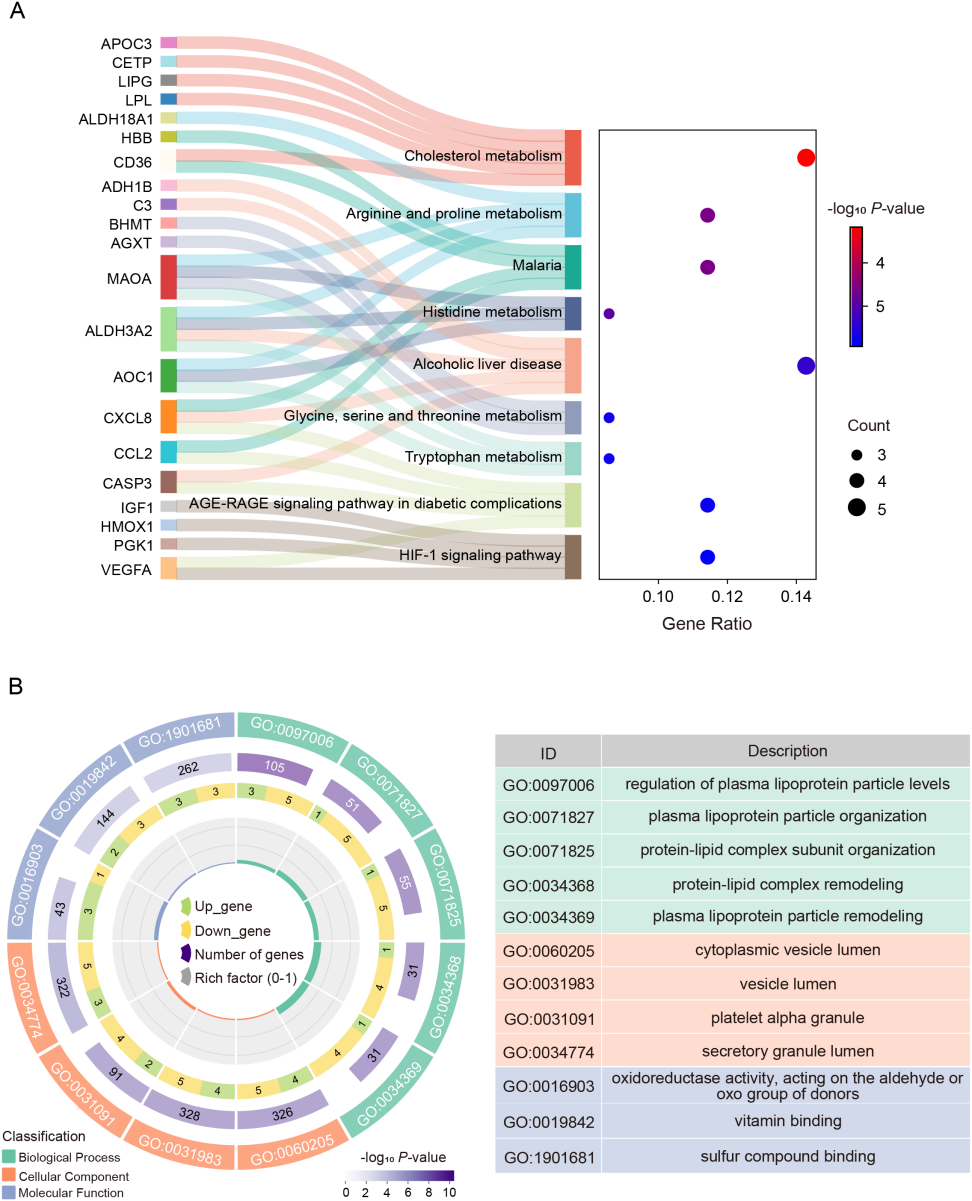
Functional and pathway enrichment analysis on 39 DE-CMGs. (A) KEGG enrichment analysis. (B) GO enrichment analysis included BP, CC, and MF terms. KEGG, Kyoto Encyclopedia of Genes and Genomes; GO, Gene Ontology; BP, biological process; CC, cellular component; MF, molecular function.

KEGG enrichment analysis of DE-CMGs was performed using DAVID database (https://davidbioinformatics.nih.gov/), mainly enriching in cholesterol metabolism, arginine and proline metabolism, histidine metabolism, glycine, serine and threonine metabolism, AGE-RAGE signaling pathway in diabetic complications and so on (Fig. 3A). Moreover, In the GO enrichment analysis, the top five BP terms predominantly centered around plasma lipoproteins, exemplified by regulation of plasma lipoprotein particle levels, plasma lipoprotein particle organization, protein-lipid complex subunit organization. For CC terms, DE-CMGs were significantly enriched in cytoplasmic vesicle lumen, vesicle lumen, platelet alpha granule, and platelet alpha granule. Furthermore, the MF terms were significantly enriched in oxidoreductase activity, acting on the aldehyde or oxo group of donors, vitamin binding, and vitamin binding (Fig. 3B).

### PPI network construction

**Figure 4:**
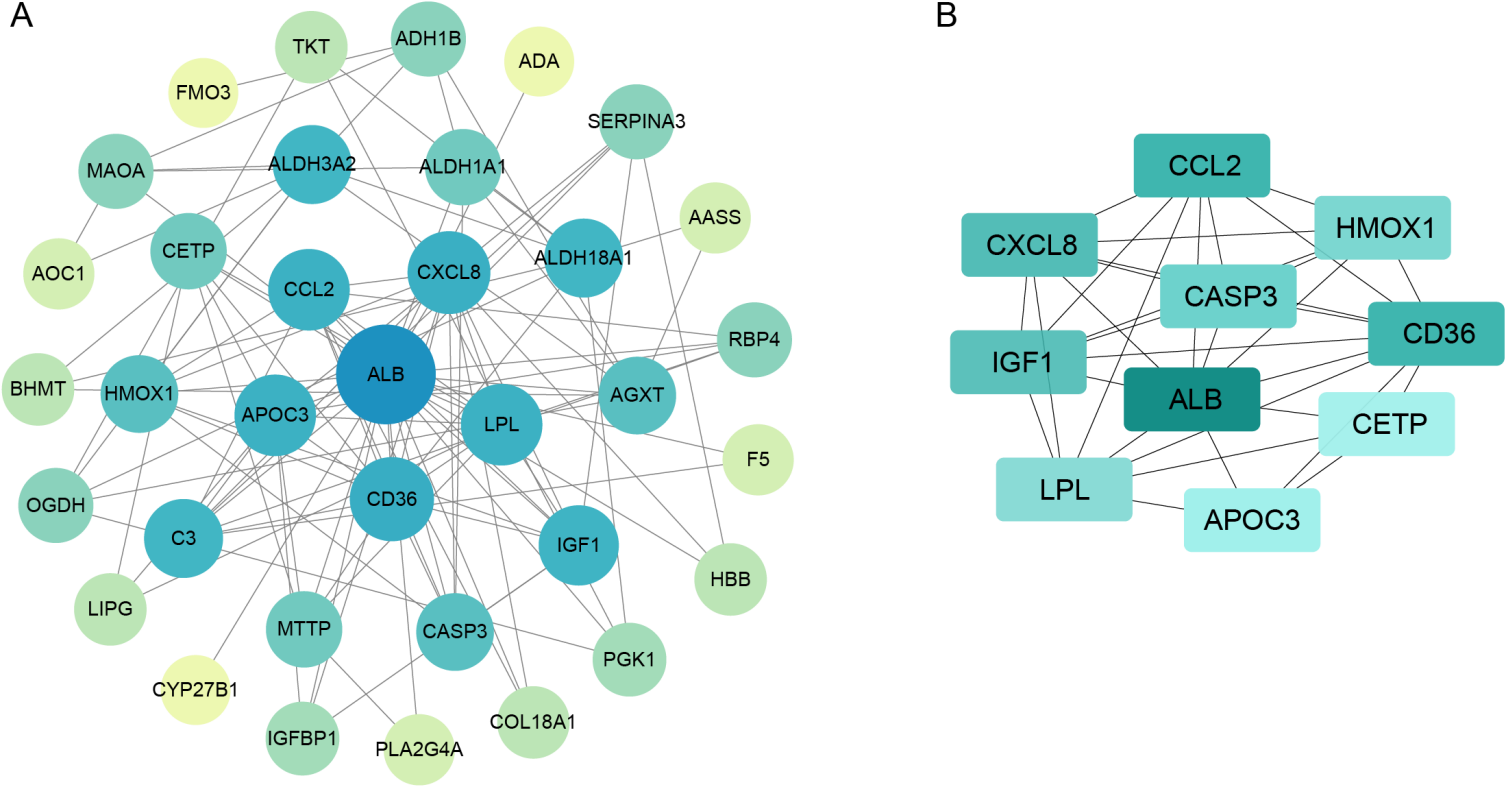
PPI network construction and candidate hub gene identification. (A) The PPI network shows interactions between DE-CMGs through nodes and edges. (B) Top 10 key genes with the highest MCC score were analyzed using the CytoHubba plugin. PPI, protein-protein interaction; MCC, maximal clique centrality.

To further investigate interactions among 39 DE-CMGs, the PPI network was constructed using the STRING database (Fig. 4A). According to MCC scores in CytoHubba, the top ten highest-scored genes were identified as candidate key genes in this study (Fig. 4B). Table 3 displays the gene list along with their names, abbreviations, and functions calculated by CytoHubba.

**Table 3:** The top 10 genes and their respective functions.

Gene	Full name	Function	UniProt ID
ALB	Albumin	Regulation of the colloidal osmotic pressure of blood	P02768
CD36	CD36 Molecule	Multifunctional glycoprotein that acts as receptor for a broad range of ligands.	P16671
CCL2	C-C Motif Chemokine Ligand 2	Acting as a ligand for C-C chemokine receptor CCR2	P13500
CXCL8	C-X-C Motif Chemokine Ligand 8	Chemotactic factor that mediates inflammatory response by attracting neutrophils, basophils, and T-cells to clear pathogens and protect the host from infection	P10145
IGF1	Insulin Like Growth Factor 1	Functionally akin to insulin, with heightened growth-promoting activity.	P05019
CASP3	Caspase 3	As a major effector caspase involved in the execution phase of apoptosis	P42574
HMOX1	Heme Oxygenase 1	Affording protection against programmed cell death	P09601
LPL	Lipoprotein Lipase	Key enzyme in triglyceride metabolism	P06858
APOC3	Apolipoprotein C3	Playing a multifaceted role in triglyceride homeostasis	P02656
CETP	Cholesteryl Ester Transfer Protein	Involved in the transfer of neutral lipids	P11597

### Identification of candidate biomarkers

**Figure 5:**
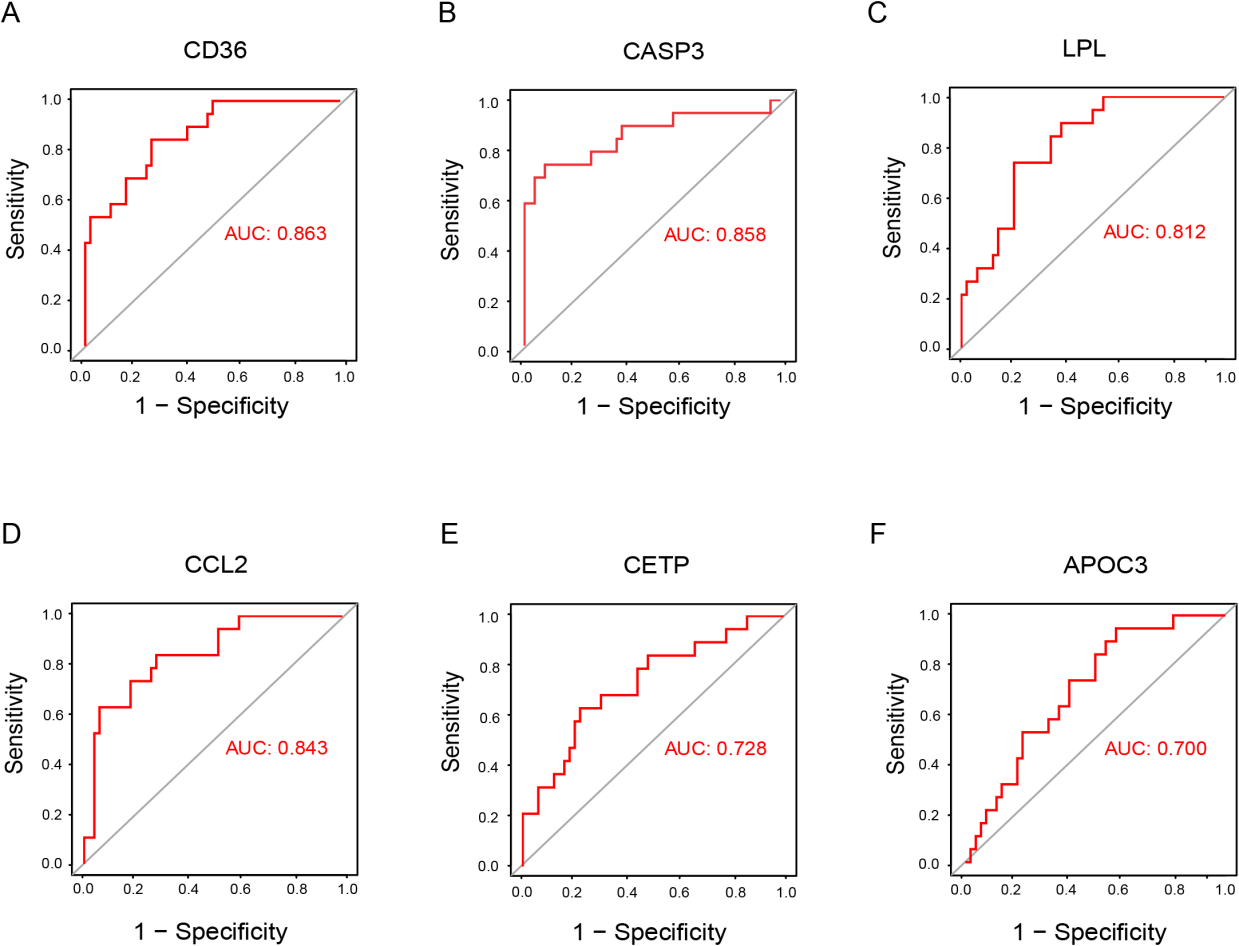
ROC curves for candidate key genes. (A) CD36. (B) CASP3. (C) LPL. (D) CCL2. (E) CETP. (F) APOC3. AUC, area under the ROC curve.

Six out of the ten DE-CMGs, including CD36, CASP3, LPL, CCL2, CETP, and APOC3, were identified as candidate key genes based on AUC > 0.7 between DN and control groups (Fig. 5). Moreover, the expression levels of these six genes were also validated using the GSE30528 and GSE30529 datasets (Fig. S2).

### The qRT-PCR experiment of biomarkers

**Figure 6:**
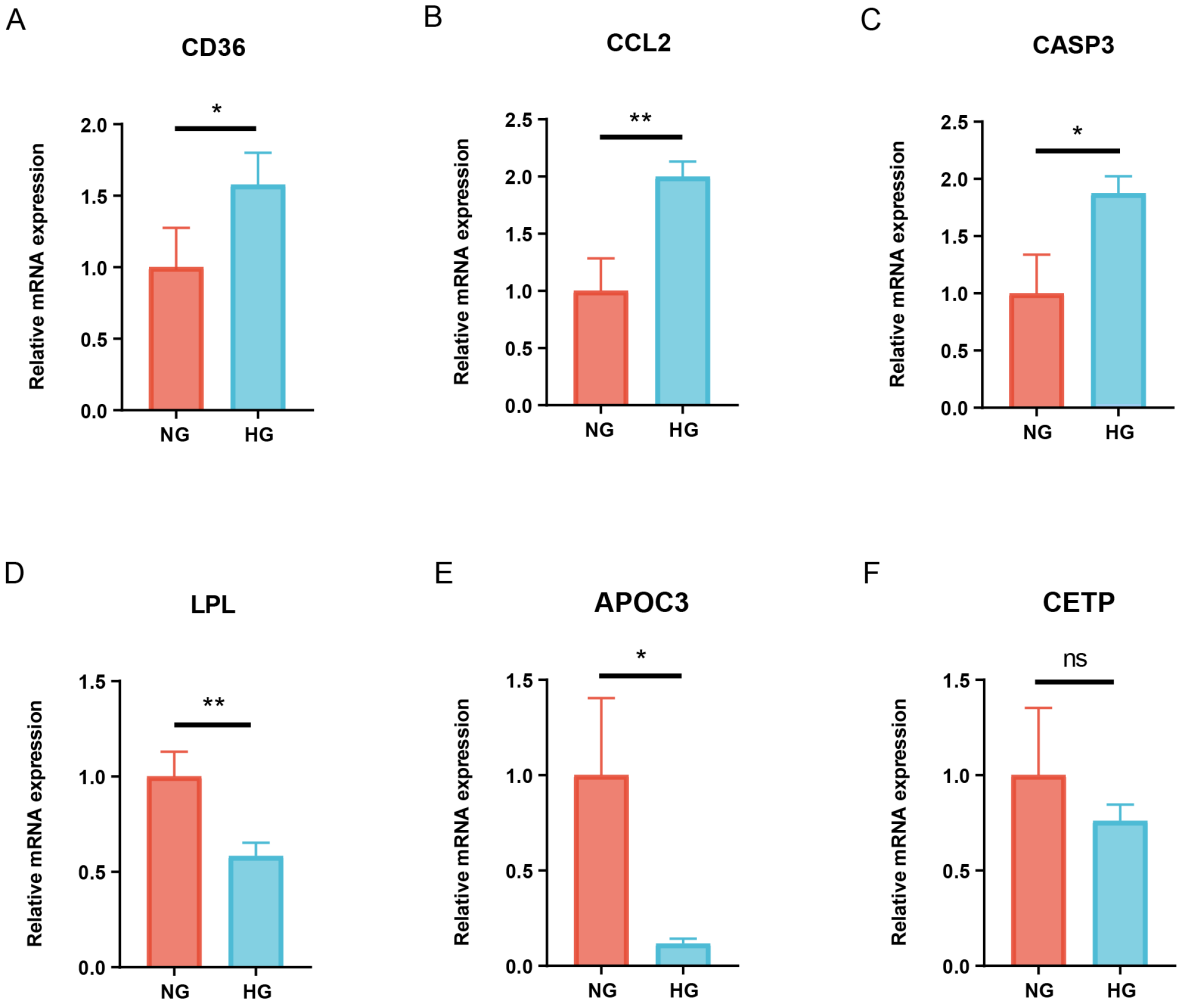
Validation of hub gene expression in high glucose-stimulated HK-2 cells by qRT-PCR. (A) CD36. (B) CCL2. (C) CASP3. (D) LPL. (E) APOC3. (F) CETP. All data were presented as the mean ± SD of triplicate experiments. HK-2 cells, human proximal tubular cells; ^∗^*P* < 0.05; ^∗∗^*P* < 0.01; ns, no statistical difference.

To further confirm the accuracy of the prediction results, the relative expression levels of six candidate biomarkers were detected through qRT-PCR experiment. The results data demonstrated that the mRNA expression level of CD36, CCL2 and CASP3 were significantly increased in the HG group compared to the NG group (*P* < 0.05, CD36, CASP3; *P* < 0.01, CCL2), whereas the expression levels of LPL and APOC3 were significantly decreased in the HG group (*P* < 0.05, APOC3; *P* < 0.01, LPL). Besides, there was no significant difference in the levels of CETP between the two groups (Fig. 6). The non-significant CETP result (*P* > 0.05) suggests it may not be a robust DN biomarker. Future studies should investigate CETP’s role in specific DN subtypes or under different glucose exposure durations to clarify its relevance.

### Model construction and potential drug prediction

**Figure 7:**
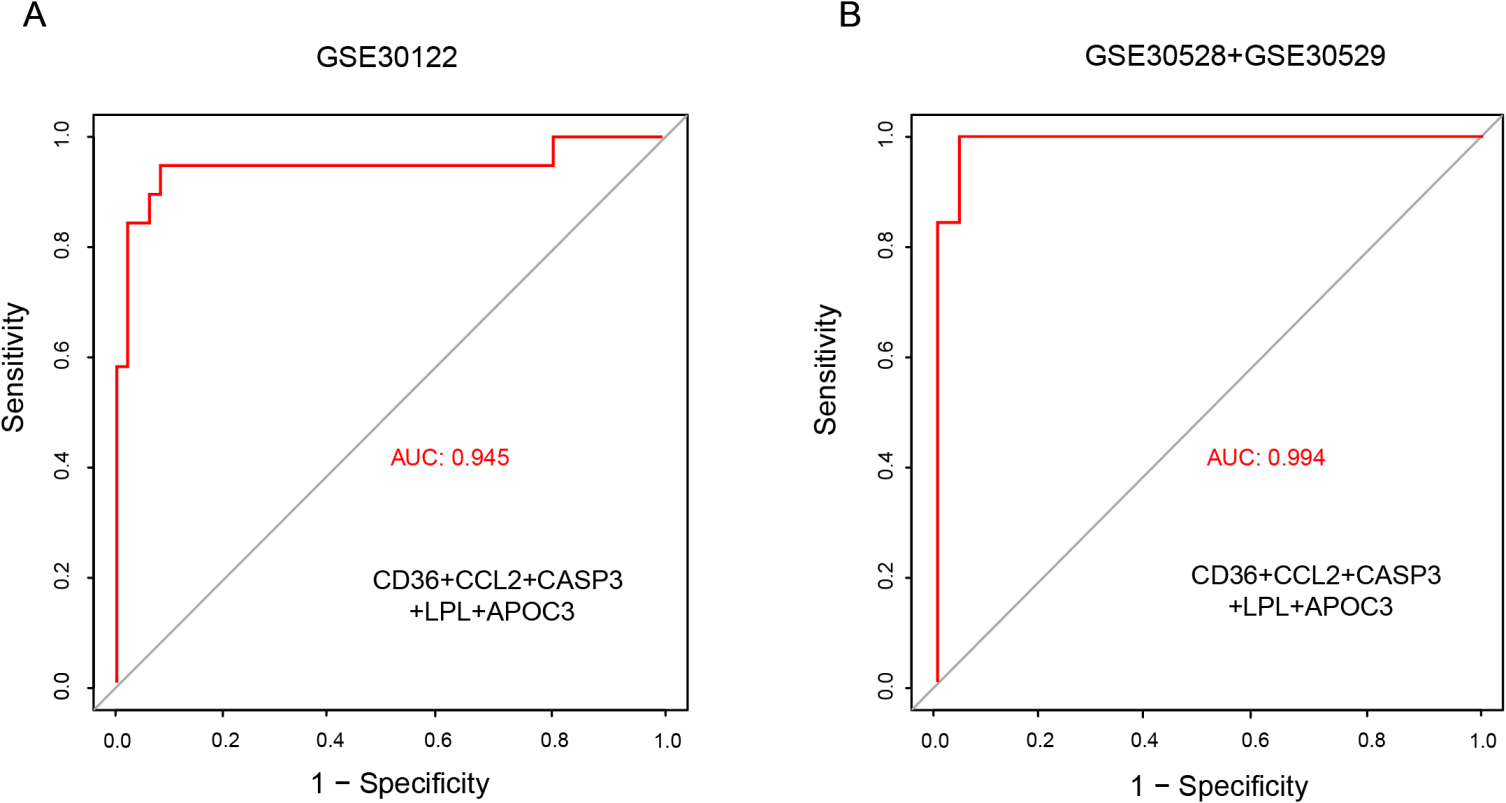
ROC curves and corresponding AUC values for combined biomarkers in DN. (A) GSE30122 training dataset (B) GSE30528 and GSE30529 test datasets.

In order to enhance diagnostic performance, a combined model based on the five validated genes was constructed, which achieved an AUC of 0.945, demonstrating good diagnostic potential (Fig. 7A). This model was subsequently validated using the combined GSE30528 and GSE30529 datasets, with an AUC of 0.994 (Fig. 7B). These findings suggest that these genes could serve as promising biomarkers for DN diagnosis. Furthermore, a total of 15 drugs targeting the biomarkers, except for CD36, were identified using the DrugBank database, with some demonstrating effectiveness in treating DN (Table 4).

**Table 4:** Small molecular therapeutic agents targeting biomarkers identified from the DrugBank database.

Gene symbol	DrugBank ID	Name	Drug group	Actions
CCL2	DB01055	Mimosine	Experimental	Inhibitor
	DB01406	Danazol	Approved	Inhibitor
	DB09301	Chondroitin sulfate	Approved, Investigational, Nutraceutical	
CASP3	DB01017	Minocycline	Approved, Investigational	Negative modulator
	DB05408	Emricasan	Investigational	
	DB13751	Glycyrrhizic acid	Approved, Experimental	Antagonist
	DB00945	Aspirin	Approved, Vet approved	Inhibitor Downregulator
	DB06255	Incadronic acid	Experimental	Activator
	DB00282	Pamidronic acid	Approved	Activator
	DB12709	Tributyrin	Investigational	Activator
	DB12843	Oleandrin	Experimental, Investigational	Regulator
LPL	DB06439	Tyloxapol	Approved, Investigational	Inhibitor
	DB09568	Omega-3-carboxylic acids	Approved, Investigational	Stimulator
	DB13751	Glycyrrhizic acid	Approved, Experimental	Inducer
APOC3	DB09130	Copper	Approved, Investigational	
	DB15067	Volanesorsen	Approved, Investigational	Inhibitor Antisense oligonucleotide

### Immune cell infiltration assessment

**Figure 8:**
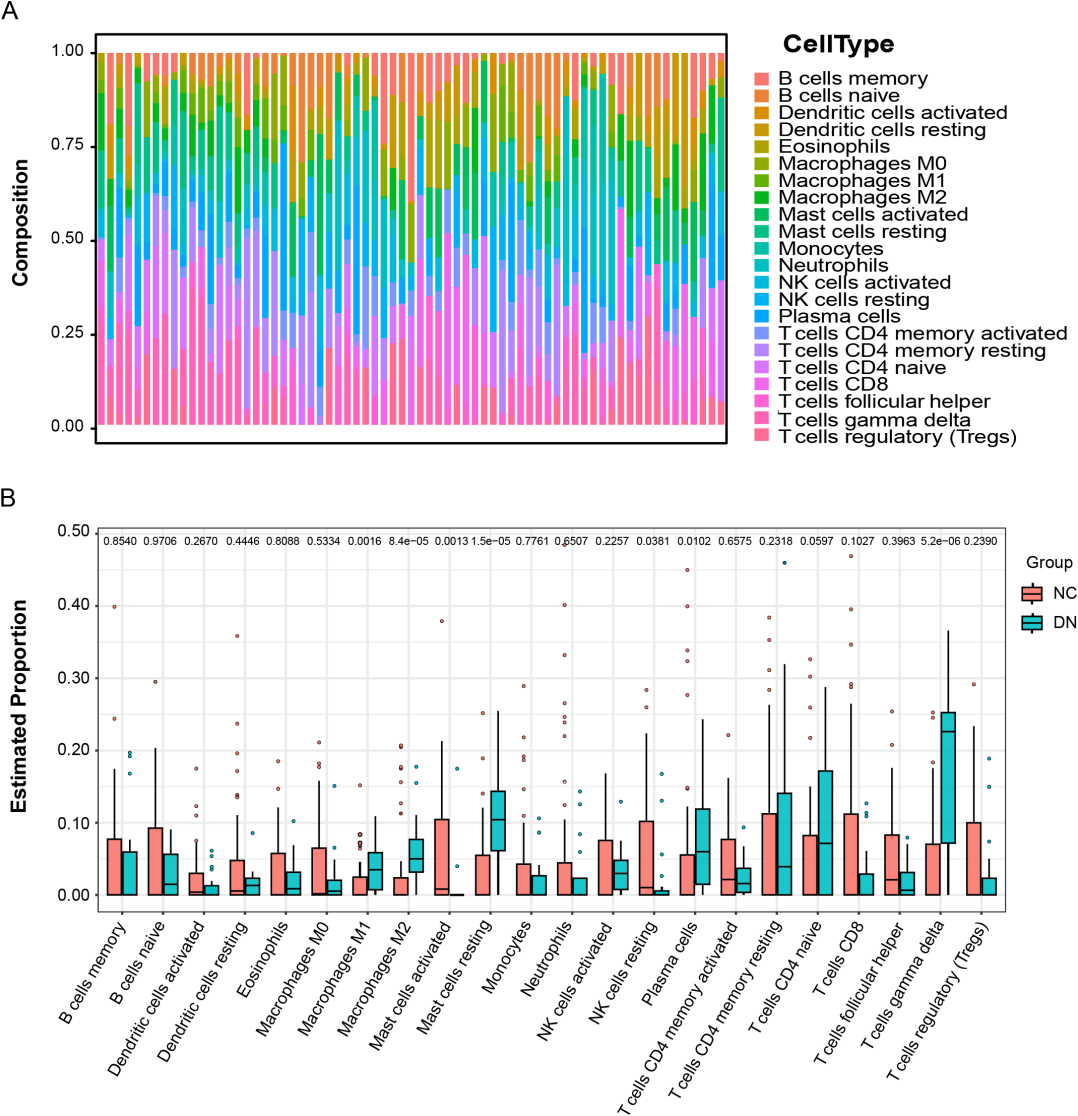
Immune cell infiltration between DN and control samples. (A) The relative percentages of 22 immune cell subpopulations in each sample (B) The difference levels of immune infiltration between DN (blue) and control (red).

The immune microenvironment consists of immune cells, extracellular matrix, various growth factors, inflammatory factors, and distinct physical and chemical characteristics, all of which significantly influence disease diagnosis and treatment sensitivity. In this study, the proportions of 22 immune cell types in 19 DN samples and 50 control samples were analyzed using the CIBERSORT algorithm. The relative abundance of immune cells in each sample is visualized in a correlation heatmap (Fig. 8A). T cells exhibited the highest expression, accounting for approximately one-third of all cells, while B cells were relatively low. Immune cell infiltration levels between DN and normal controls were compared in a boxplot (Fig. 8B). The DN group showed significantly higher proportions of M1 macrophages (*P* = 0.0016), M2 macrophages (*P* = 8.4e−05), resting mast cells (*P* = 1.5e−05), plasma cells (*P* = 0.0102), and T cells gamma delta (*P* = 5.2e−06) compared to controls. Conversely, activated mast cells (*P* = 0.0013) and resting NK cells (*P* = 0.0381) were significantly lower in the DN group compared to controls.

**Figure 9:**
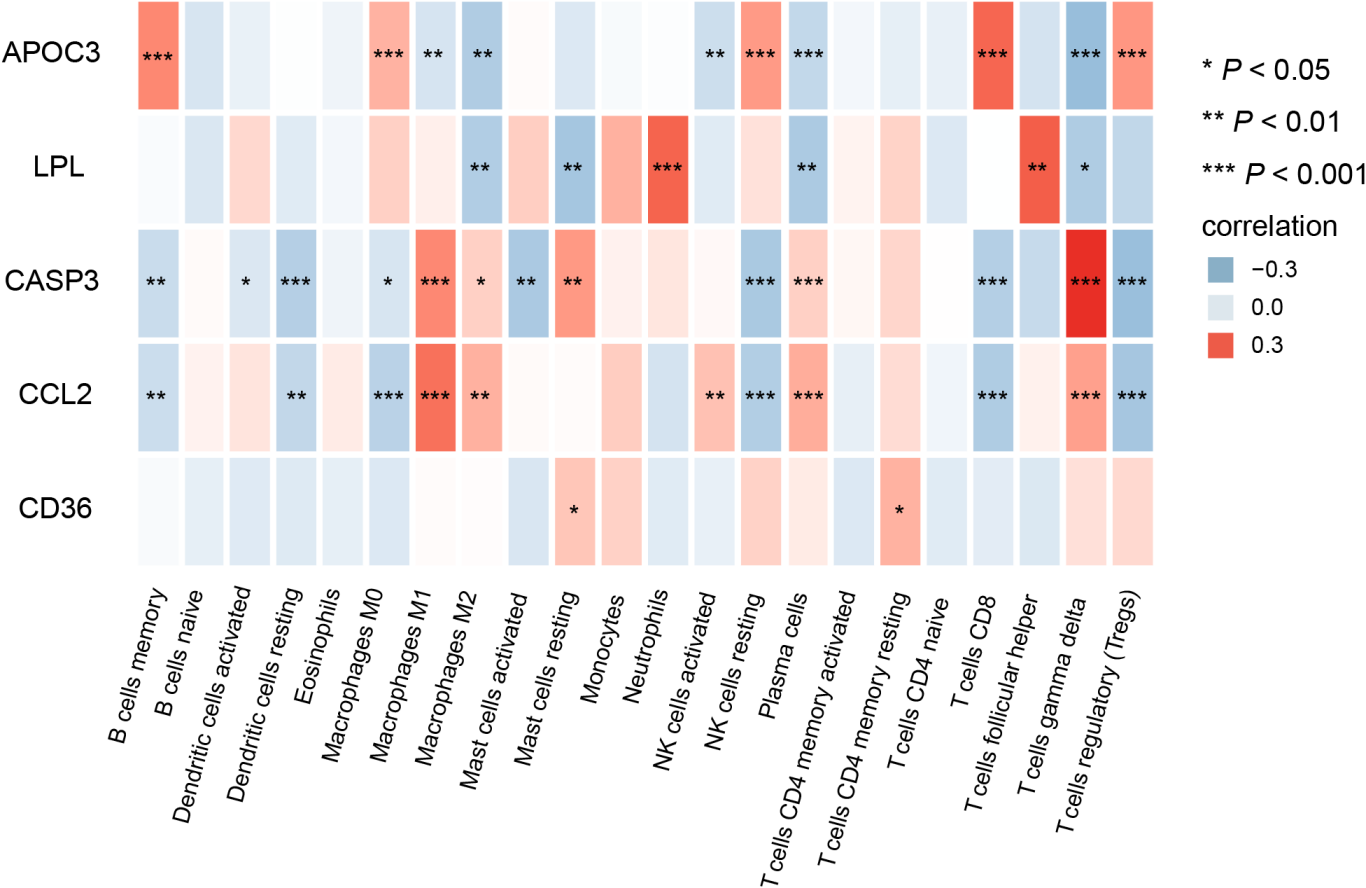
Correlation between the five hub genes and immune infiltrating cells (^∗^*P* < 0.05, ^∗∗^*P* < 0.01, ^∗∗∗^*P* < 0.001).

Subsequently, the relationship between the validated five genes and immune infiltration was investigated. The five genes demonstrated significant negative associations with resting dendritic cells, T cells, NK cells, and macrophages M0. In contrast, positive associations were observed with macrophages M1, neutrophils, and T cells gamma delta (Fig. 9). These findings suggest that the hub genes may play a pivotal role in modulating the immune microenvironment.

### Clinical correlation analysis

**Figure 10:**
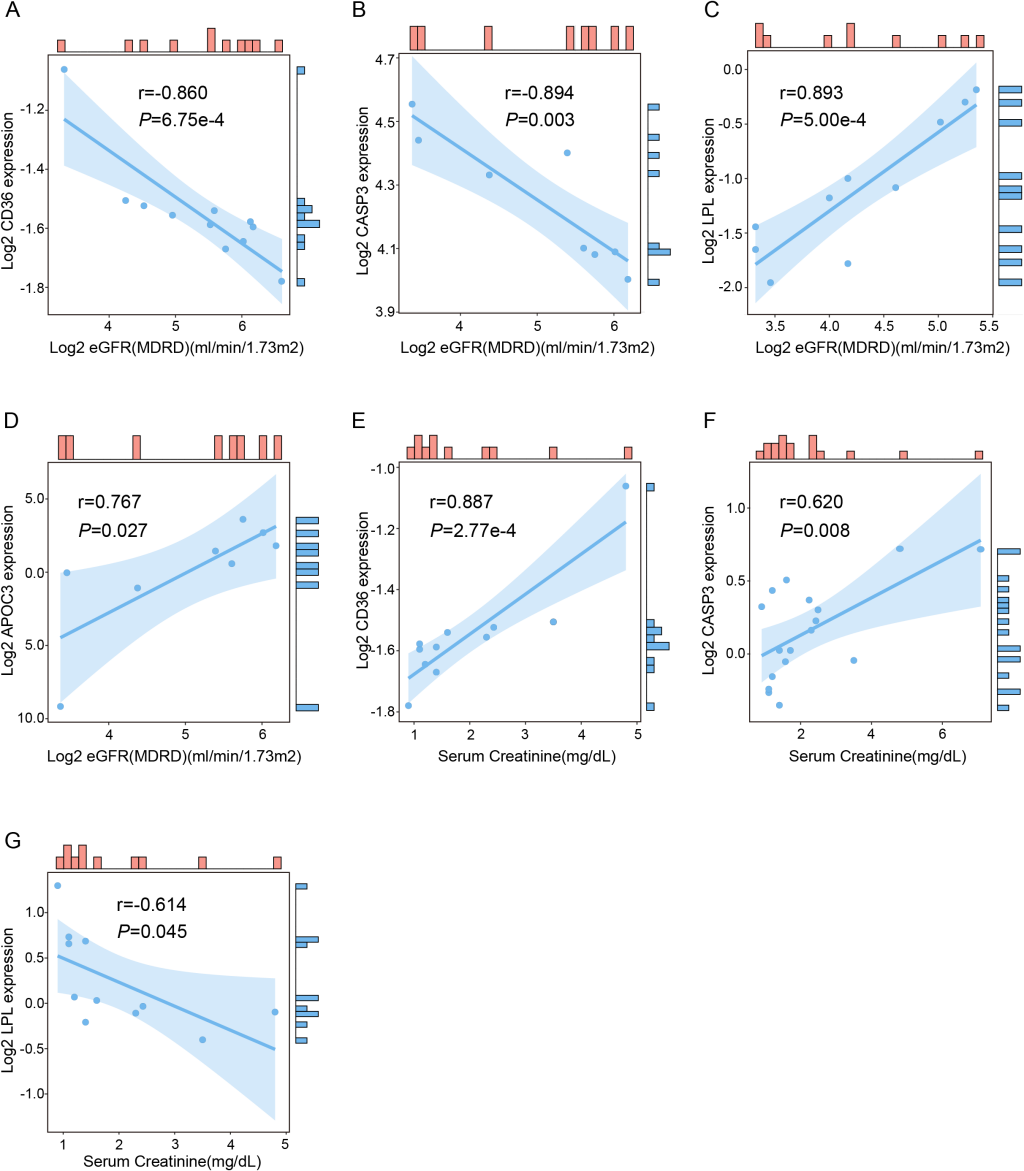
Correlation of hub gene expression levels with clinical renal function indicators. (A–D) Correlation analysis of CD36, CASP3, LPL and APOC3 with eGFR, assessed using the modification of diet in renal disease (MDRD) equation. (E–G) Correlation analysis of CD36, CASP3 and LPL with serum creatinine. eGFR, estimated glomerular filtration rate.

The estimated glomerular filtration rate (eGFR) and serum creatinine, crucial for assessing renal function, are pivotal in monitoring the progression and severity of renal disorders, including diabetic nephropathy. Correlation analysis revealed strong negative associations between the expressions of CD36 (*r* = −0.860, *P* < 0.001) and CASP3 (*r* = −0.894, *P* < 0.01) with eGFR. Conversely, positive correlations were observed between LPL (*r* = 0.893, *P* < 0.001) and APOC3 (*r* = 0.767, *P* < 0.05) expressions with eGFR. Additionally, positive associations were found between the expressions of CD36 (*r* = 0.887, *P* < 0.001) and CASP3 (*r* = 0.620, *P* < 0.01) with serum creatinine levels. Conversely, negative correlations were noted between the expressions of LPL (*r* = −0.614, *P* < 0.05) and serum creatinine levels (Fig. 10).

## Discussion

Copper is essential for maintaining mammalian health, playing a critical role in various physiological processes (Kong & Sun, 2023). Both dietary copper intake and plasma copper levels have been suggested to have a potential link with the onset of diabetes (Stanikowski et al., 2020). Furthermore, recent studies have indicated that an imbalance in copper levels can contribute to the advancement of DN and disrupt antioxidant homeostasis (Gembillo et al., 2022; Takao et al., 2022). Copper dysregulation may promote cuproptosis, enhancing mitochondrial dysfunction and inflammation in DN, as evidenced by the upregulation of CD36 and CASP3.

In this study, the 833 DEGs obtained from the GSE30122 dataset in the GEO database were intersected with 388 CMGs selected by GeneCards, resulting in the identification of 39 DE-CMGs. Comprehensive GO and KEGG enrichment analyses unveiled that these DE-CMGs predominantly participate in regulating plasma lipoprotein levels and cellular amino acid processes, both of which are associated with the pathology of DN. Subsequently, the top ten DE-CMGs were further screened using CytoHubba, and six genes (CD36, CCL2, CASP3, LPL, APOC3, and CETP) with an AUC > 0.7 were identified as candidate biomarkers.

In addition, the qRT-PCR experiment revealed significant differences in the expression of five genes between the HG and NG groups in the HK-2 cell model. Although CETP decreased in the HG group, there was no significant difference in the change between the groups. Therefore, CETP was excluded from further analysis in this study, which focused on establishing a diagnostic model based on genes with significant expression changes. Further studies are required to explore the mechanism underlying its non-significant alteration. The combination of these five genes exhibited good diagnostic performance, with an AUC value reaching as high as 0.945. CD36, serving as a receptor for a broad spectrum of ligands, has been found to partially alter cellular metabolic pathways in diabetic kidney disease (Niu et al., 2023). CCL2, a ligand for C-C chemokine, contributes to glomerulosclerosis and mediates macrophage-associated inflammation (Darisipudi et al., 2011). CASP3, a key executor of apoptosis, has been consistently implicated in the pathogenesis of DN (Wen et al., 2020). LPL, the key enzyme in triglyceride metabolism, potentially accelerates renal function decline in type 2 diabetes patients (Wu et al., 2023). APOC3, which plays a multifaceted role in triglyceride homeostasis, has been reported to exacerbate early-stage diabetic nephropathy by activating the renal TLR2/NF-κB pathway (Wang et al., 2021). Moreover, the expression levels of CD36 and CASP3 show a negative correlation with eGFR, while LPL and APOC3 correlate positively with eGFR. CD36 and CASP3 also exhibit a positive correlation with serum creatinine levels, whereas LPL shows a negative correlation. The above results indicate that these five genes may serve as potential diagnostic and prognostic biomarkers to differentiate DN from normal individuals.

Substantial evidence underscores the pivotal involvement of the immune system in the pathogenesis of DN (Wu & Humphreys, 2022). Immune infiltration analysis revealed significant differences between control and DN samples in multiple immune cell types, including M1 and M2 macrophages, resting and activated mast cells, plasma cells, gamma delta T cells, and natural killer (NK) cells. Besides, the identified hub genes exhibited significant correlations with these immune cells, suggesting a potential role in immune-related mechanisms of DN. M1 macrophages, prevalent in DN, initiate inflammation, tissue damage, and renal fibrosis *via* proinflammatory cytokines such as TNF-a, IL-6, IL-10, and monocyte chemoattractant protein 1 (MCP-1) (Kim et al., 2015; Wang et al., 2017). Conversely, M2 macrophages aid in wound healing by producing anti-inflammatory and proangiogenic factors. A study suggests that targeting the RAS pharmacologically reduces albuminuria, improves renal function trajectory, diminishes kidney macrophage infiltration, and promotes the transition of macrophage phenotype from M1 to M2 (Moratal et al., 2021). Mast cells, primarily located in peritubular, perivascular, and periglomerular interstitial regions, release bioactive substances such as tryptase, chymase, and TNF-a, thus contributing to renal inflammation, fibrosis, and DN progression (Zheng et al., 2012). Plasma cells can manifest various renal complications due to damage caused by circulating light and heavy-chain immunoglobulin components (Herrera, 2009). T cells were observed infiltrating kidney biopsies in individuals with DN (Forbes et al., 2021), while studies on the role of NK cells in DN are scarce.

The findings of this study carry substantial implications for advancing the development of novel diagnostic biomarkers and actionable targets, thereby broadening the scope of treatment options available for patients with DN. However, the present study encountered certain limitations, notably the absence of crucial clinical information such as diabetes type, hypertension, and dyslipidemia, which could potentially influence gene expression profiles and the identification of hub genes. Additionally, the reliance on a single cell line (HK-2) limits insights into podocyte or mesangial cell contributions to DN. Moreover, considering larger numbers of DN patient samples is essential to validate the stability of the research results. Therefore, further studies *in vivo* and *in vitro* are necessary in the future.

## Conclusions

This study underscores the pivotal role of copper metabolism in DN by identifying five hub genes (CD36, CCL2, CASP3, LPL, APOC3) as potential diagnostic biomarkers. These genes, linked to oxidative stress, inflammation, and lipid metabolism, offer insights into DN’s pathogenesis and its immune microenvironment, particularly through correlations with M1/M2 macrophages and T cells. The strong association of these genes with clinical markers like eGFR suggests their utility in early DN detection. However, limitations such as the lack of clinical data on diabetes type and reliance on a single cell line (HK-2) necessitate caution in generalizing findings. Future research should validate these biomarkers in clinical cohorts, explore copper-induced cuproptosis mechanisms in DN animal models, and test the efficacy of identified therapeutic agents in clinical trials. These findings pave the way for novel diagnostic and therapeutic strategies targeting copper metabolism to mitigate DN progression.

##  Supplemental Information

10.7717/peerj.20468/supp-1 Supplemental Information 1 Principal Component Analysis (PCA) of the merged GSE30528 and GSE30529 datasets before and after batch effect correction (A) PCA of the originally merged datasets before batch-corrected . (B) PCA of the batch-corrected merged datasets.

10.7717/peerj.20468/supp-2 Supplemental Information 2 Expression of candidate hub genes in the DN and control groups (A) GSE30528 dataset (B) GSE30529 dataset.

10.7717/peerj.20468/supp-3 Supplemental Information 3 Gene expresssion in GSE30122

10.7717/peerj.20468/supp-4 Supplemental Information 4 833 DEGs of GSE30122

10.7717/peerj.20468/supp-5 Supplemental Information 5 The expression levels of the DE-CMGs in GSE30122

10.7717/peerj.20468/supp-6 Supplemental Information 6 The results of Gene Ontology (GO) enrichment analysis

10.7717/peerj.20468/supp-7 Supplemental Information 7 The results of Kyoto Encyclopedia of Genes and Genomes (KEGG) enrichment analysis

10.7717/peerj.20468/supp-8 Supplemental Information 8 qRT-PCR _data

10.7717/peerj.20468/supp-9 Supplemental Information 9 MIQE checklist

10.7717/peerj.20468/supp-10 Supplemental Information 10 Code

## Additional Information and Declarations

### DNA Deposition

The following information was supplied regarding the deposition of DNA sequences: The sequences in this study are available at GEO: GSE30122, GSE30528, GSE30529.

### Data Availability

The following information was supplied regarding data availability: The raw data are available in the Supplemental Files. The dataset is available at GeneCards: https://www.genecards.org/
- The dataset is hosted in a restricted-access repository. To download it, users must register for a free account on the platform. Users must search “copper metabolism” after logging in. The dataset is available at Nephroseq v5: https://www.nephroseq.org/
- The dataset is hosted in a restricted-access repository. To download it, users must register for a free account on the platform using academic credentials. Key genes: CD36, CASP3, LPL, APOC3, and CCL2 The sequences in this study are available at GEO: GSE30122, GSE30528, GSE30529.
