# Supplementary figures and images for: Copper metabolism-related biomarkers and therapeutic targets for diabetic nephropathy
Source: PeerJ. 2025 Dec 19;13:e20468. doi: 10.7717/peerj.20468 (PMC12721120; doi:10.7717/peerj.20468)

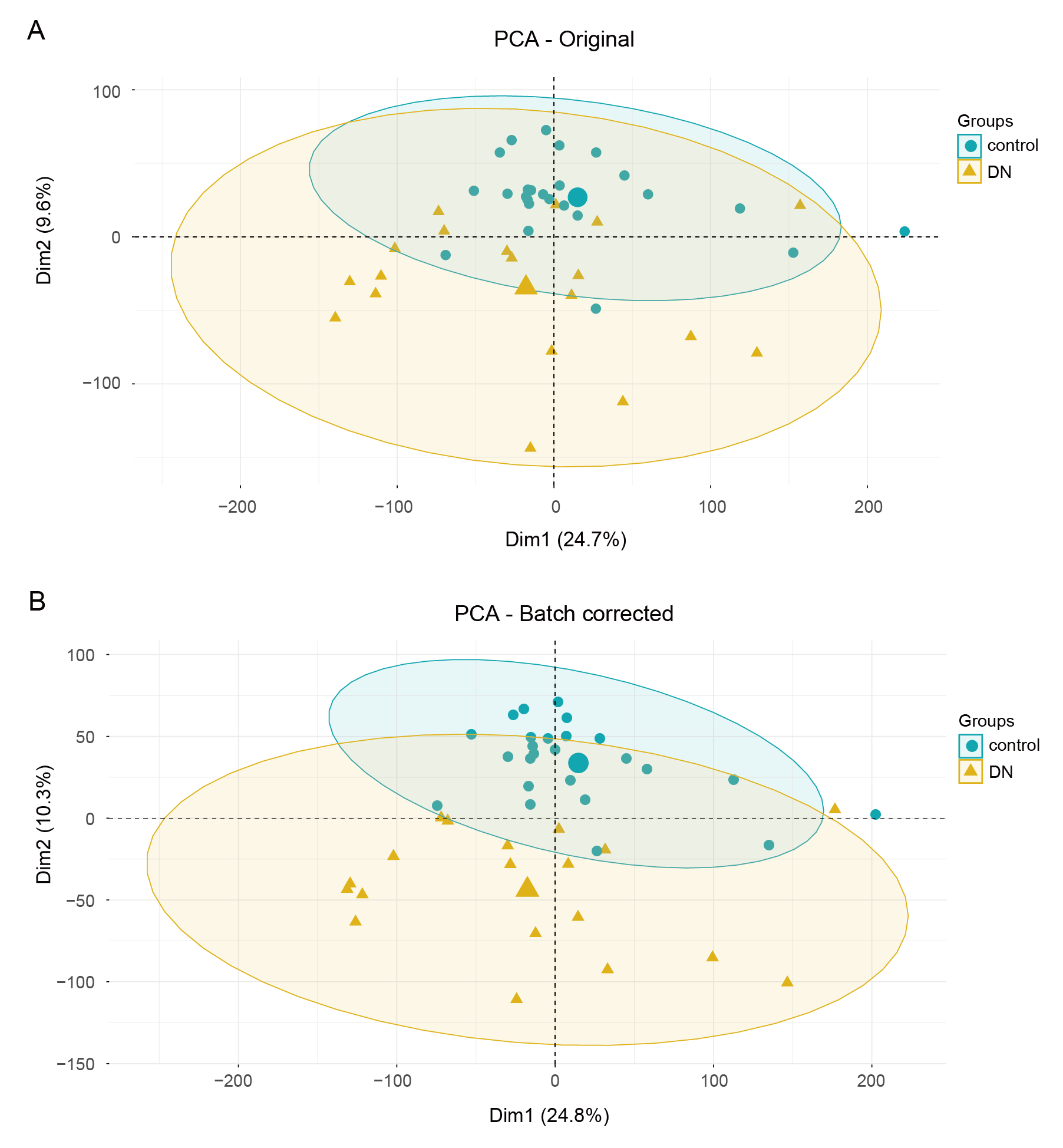

Supplement: Supplemental Information 1 — (A) PCA of the originally merged datasets before batch-corrected . (B) PCA of the batch-corrected merged datasets. [file peerj-13-20468-s001.png]

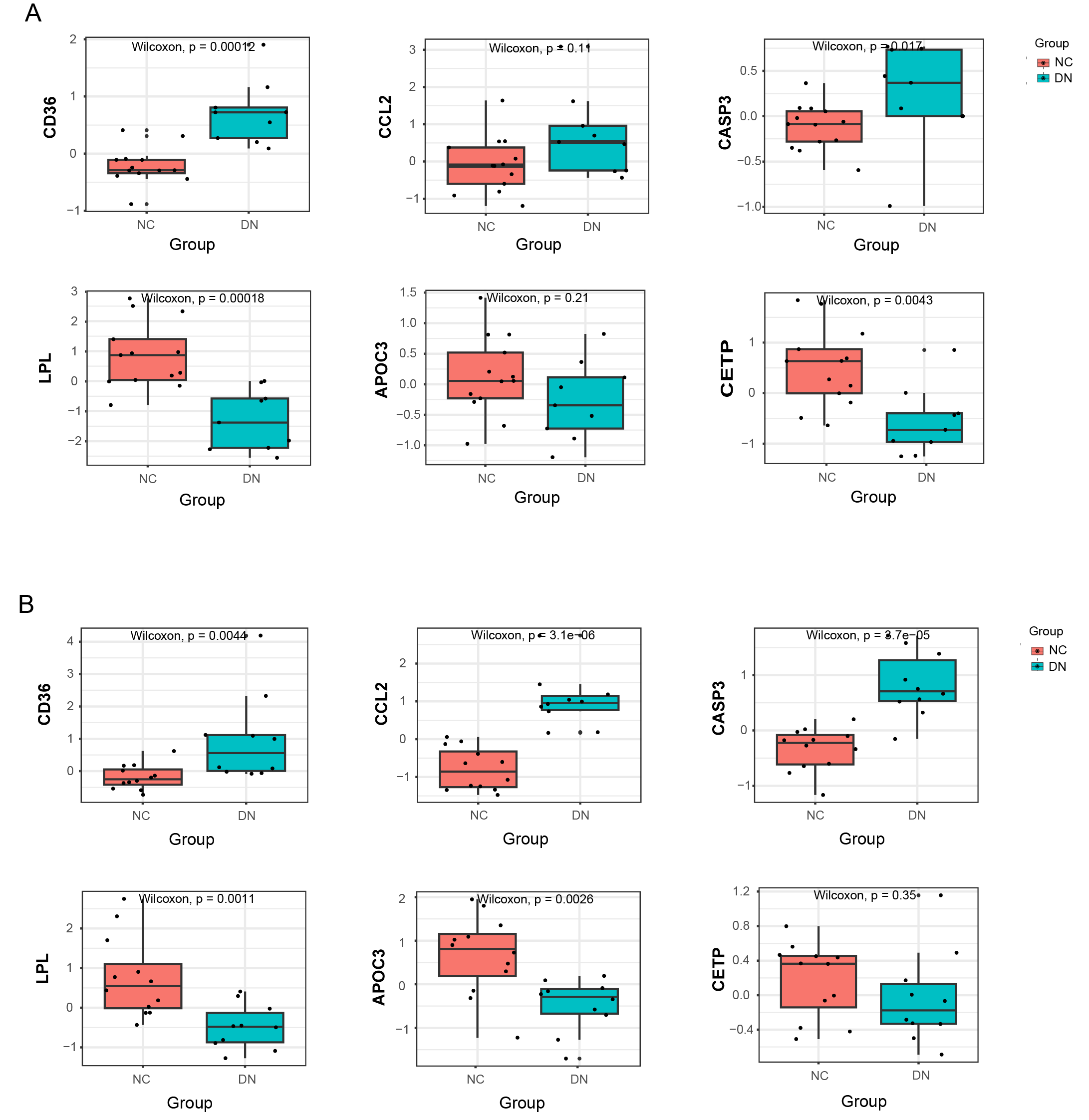

Supplement: Supplemental Information 2 — (A) GSE30528 dataset (B) GSE30529 dataset. [file peerj-13-20468-s002.png]
